# Immunogenicity of three doses of anti-SARS-CoV-2 BNT162b2 vaccine in psoriasis patients treated with biologics

**DOI:** 10.3389/fmed.2022.961904

**Published:** 2022-09-06

**Authors:** Dario Graceffa, Francesca Sperati, Claudio Bonifati, Gabriele Spoletini, Viviana Lora, Fulvia Pimpinelli, Martina Pontone, Raul Pellini, Ornella Di Bella, Aldo Morrone, Antonio Cristaudo

**Affiliations:** ^1^Department of Clinical Dermatology, San Gallicano Dermatological Institute, IRCCS, Rome, Italy; ^2^Biostatistics Unit, San Gallicano Dermatological Institute IRCCS, Rome, Italy; ^3^General Surgery and Liver Transplantation, Fondazione Policlinico Universitario Agostino Gemelli IRCCS, Rome, Italy; ^4^Microbiology and Virology Unit, Dermatological Clinical and Research Department, San Gallicano Dermatological Institute, IRCCS, Rome, Italy; ^5^Department Otolaryngology Head and Neck Surgery, IRCCS Regina Elena National Cancer Institute, Rome, Italy; ^6^Medical Direction, IRCCS Regina Elena National Cancer Institute, Rome, Italy; ^7^Scientific Direction, San Gallicano Dermatological Institute, IRCCS, Rome, Italy

**Keywords:** psoriasis, vaccine, immunogenicity, biologics, COVID-19

## Abstract

**Introduction:**

Psoriasis has not been directly linked to a poor prognosis for COVID-19, yet immunomodulatory agents used for its management may lead to increased vulnerability to the dangerous complications of SARS-CoV-2 infection, as well as impair the effectiveness of the recently introduced vaccines. The three-dose antibody response trend and the safety of BNT162b2 mRNA vaccine in psoriasis patients treated with biologic drugs have remained under-researched.

**Materials and methods:**

Forty-five psoriatic patients on biologic treatment were enrolled to evaluate their humoral response to three doses of BNT162b2. IgG titers anti-SARS-CoV-2 spike protein were evaluated at baseline (day 0, first dose), after 3 weeks (second dose), four weeks post-second dose, at the time of the third dose administration and 4 weeks post-third dose. Seropositivity was defined as IgG ≥15 antibody-binding units (BAU)/mL. Data on vaccine safety were also collected by interview at each visit.

**Results:**

A statistically significant increase in antibody titers was observed after each dose of vaccine compared with baseline, with no significant differences between patients and controls. Methotrexate used in combination with biologics has been shown to negatively influence the antibody response to the vaccine. On the contrary, increasing body mass index (BMI) positively influenced the antibody response. No adverse effects were reported, and no relapses of psoriasis were observed in the weeks following vaccine administration in our study population.

**Conclusions:**

Our data are largely consistent with the recent literature on this topic confirming the substantial efficacy and safety of BNT162b2 mRNA vaccine on psoriatic patients treated with biologics of different types and support the recommendation to perform additional doses in this specific subgroup of patients.

## Introduction

The 2019 coronavirus disease (COVID-19) significantly impacted patients with chronic autoimmune diseases also because of their concomitant immunomodulating treatments. Psoriasis and immunomodulators have been linked to an increased risk of serious infections, including viral pneumonias (1–7).

In addition, psoriasis patients frequently suffer from cardio-metabolic comorbidities, now considered as strong risk factors for acute respiratory distress syndrome (ARDS) and poor COVID-19 prognosis (8–10).

However, preliminary data from several large cohort studies assessing the risk of hospitalization, intensive care unit admission, and mortality due to COVID-19 in psoriasis patients treated with systemic immunomodulating therapies showed only minimal increased risk of COVID-19-related complications and poor prognosis mainly in individuals on conventionals immunomodulators such as Methotrexate (11–16).

Previous evidence suggests immunomodulators may prevent the hyperactivity of the innate immune system underlying the cytokine storm and subsequent multi-organ damage induced by SARS-CoV-2 infection (17–19).

Nevertheless, several scientific societies and the Italian Ministry of Health, in view of a potential immunological vulnerability, included psoriasis patients currently on immunomodulatory treatment, among the high-priority categories for COVID-19 vaccination.

To date, four COVID-19 vaccines are authorized by EMA for public use (20–25): two mRNA vaccines (Pfizer–BioNTech BNT162b2 and Moderna mRNA-1273), and two viral vector vaccines (Oxford–AstraZeneca AZD1222 and Johnson & Johnson Ad26.CoV2.S).

Our institution implemented a priority-based vaccination plan with Pfizer-BioNTech vaccine for obese or immunocompromised patients. BNT162b2 is a nucleoside-modified mRNA vaccine encoding the SARS-CoV-2 spike glycoprotein and it is administered intramuscularly as two doses given 21 days apart.

Although BNT162b2 was found to be 95% effective in preventing COVID-19 in global phase 2 and 3 studies (26), several concerns have been raised and investigated in several clinical trials and reports around impaired protective response (27–37) and psoriasis relapses after vaccine administration (38–54).

National Psoriasis Foundation COVID-19 Task Force provides guidance for the management of patients with psoriatic disease during the pandemic and strongly encourages patients who do not have contraindications to vaccination, to receive an mRNA-based COVID-19 regardless of the concurrent use of therapies for psoriasis and/or psoriatic arthritis (55). Conversely, the American College of Rheumatology, recommended to withhold methotrexate 1 week after each dose of vaccine for patients with well-controlled disease, basing this recommendation on data from influenza and pneumococcal vaccines (56).

Although testing for vaccine immune response is not currently recommended, many studies have estimated the vaccine's protective effectiveness on the basis of its ability to induce a humoral response (57–59).

We assume that post-vaccine IgG titer might be used as a reliable surrogate to predict vaccine efficacy.

At present, only limited data are available on the antibody response to COVID-19 mRNA vaccines in psoriasis patients, particularly regarding their trend after each dose. The present study aims to assess the humoral responses to three doses of SARS-CoV-2 mRNA vaccine BNT162b2 in a cohort of psoriasis patients on biologic treatment.

## Materials and methods

A prospective single-center cohort study was conducted to assess the immunogenicity and safety of three doses of BNT162b2 on psoriatic patients treated with biologic drugs. From April 1 to May 15, 2021, 45 psoriasis patients receiving biologics, obese (BMI ≥30 kg/m^2^) or with HIV infection as additional risk factor for immune impairment, were enrolled and followed prospectively for nine months. A group of 45 healthy controls matched by age, sex, and BMI were recruited from the staff of the Istituti Fisioterapici Ospitalieri (IFO), Rome, Italy for comparison of the antibody responses.

The vaccination schedule was based on two intramuscular injections of 30 μg per dose of BNT162b2 vaccine 3 weeks apart, followed by a third dose administered 5 months apart from the second dose. Neither suspension nor dose modification of the biologic therapy schemes was planned in any treatment set, whereas in accordance with the recommendations of the American College of Rheumatology, patients were advised to discontinue methotrexate for 1 week after each dose of vaccine.

Neutralizing IgG titers anti-SARS-CoV-2 spike protein were evaluated at baseline (day 0, first injection, time point TP0), after 3 weeks (day 21, second injection, TP1), four weeks post-second dose (day 51, TP2), at the time of the third dose administration (day 200, TP3) and four weeks post-third dose (day 230, TP4).

Dermatologic and rheumatologic assessments were carried out at baseline and data on vaccine safety were also collected on TP1, TP2 and TP4 by interview. Local and systemic side effects as well as any flare-up of the underlying disease occurred after vaccine administration were recorded. All participants were asked to provide nose and throat swabs on each defined TP but no antibody tests to detect any asymptomatic SARS-CoV-2 infections were performed on the study population.

The study was approved by the IRCCS Central Ethical Committee of Regione Lazio in January 2021 (Prot. N-1463/21) and conducted in compliance with the Helsinki Declaration and Good Clinical Practice. All subjects signed a specific written informed consent before study enrollment.

Serological test and definition LIAISON^®^ SARS-CoV-2 S1/S2 IgG by DiaSorin^®^, Saluggia, Italy. The LIAISON^®^ SARS-CoV-2 S1/S2 IgG test is a quantitative chemiluminescent immunoassay (CLIA), fully automated on LIAISON^®^ XL platform, for the detection of IgG antibodies against the subunits S1 and S2 of SARS-CoV-2 spike protein. The subunits S1 and S2 are responsible for binding and fusion of virus to the host cell, respectively, and are both targets of neutralizing antibodies. According to the manufacturer technical manual^1^, the result of a LIAISON^®^ SARS-CoV-2 S1/S2 IgG test has to be reported as positive with a signal of 15 AU/mL or higher, equivocal between 12 and 15 AU/mL and negative and <12 AU/mL.

Clinical and analytic performance of this automated serological test identifying SARS-CoV-2 S1/S2-neutralizing IgG in a semi-quantitative manner was published in September 2020 (60).

For the analysis' purposes, the result of 15 AU/mL indicated by DiaSorin^®^ was considered as the cutoff to discriminate responders from non-responders to vaccination.

### Statistical analysis

To control for potential confounders that could affect the outcomes of interest, propensity score matching (PSM) was employed (61, 62) to generate two different groups of patients (i.e., affected and not affected by psoriasis) with balanced distribution of baseline features. Presence of psoriasis was considered as dependent variable, with the absence of psoriasis as control group. Covariates included in the analysis were age, gender and BMI. Patients and controls were matched 1:1 with the nearest-neighbor method and using no caliper distance of the standard deviation of the logit of the estimated propensity score to ensure good matches. Balance between the two groups was assessed using the relative multivariate imbalance measure L1 proposed by Iacus, King and Porro (63, 64).

We reported the categorical variables through absolute and relative frequencies, whereas the continuous variables through means with standard deviations (SD). Geometric mean of AU/mL concentration (GMC) and its 95% confidence interval (95%CI) was reported for all time points and for each group. TP4/TP3 and TP2/TP1 ratio were computed and reported using the geometric mean (GM) and its 95%CI. Kolmogorov-Smirnov normality test was calculated for all the continuous variables. To explore the differences between continuous variables, Mann-Whitney or the T-Student test were utilized, according to the nature of data distribution. The relationships between categorical variables were analyzed using the Pearson's Chi-square. The Wilcoxon test was applied to compare the IgG titer at TP4, TP3, T2 and T1 with the baseline value in psoriasis patients and control subjects. A generalized univariate and multivariate linear model (GLM) was implemented to evaluate the correlation between logarithm of IgG titer at TP4 and covariates. *P*-values < 0.05 were considered significant. All statistical analyses were performed using SPSS statistical software version 21 (SPSS inc., Chicago IL, USA).

### Propensity score matched analysis

PSM was identified in 90 (45 per group) matched patients at a 1:1 ratio out of a total of 128 patients. The L1 test measure was larger in the unmatched sample (0.931) than in the matched sample (0.911), indicating that the two groups were well balanced across all variables considered. The absence of differences between the two groups regarding patient gender confirmed the success of the matching for this variable. BMI and age remained statistically different due to the substantial gap in values between the two groups before the matching.

## Results

### Enrollment and characteristics of the study population

Out of 700 psoriasis patients on biologic treatment followed at our Psoriasis Clinic, 45 patients (28 males and 17 females) matched the inclusion criteria and were enrolled in the study. Baseline characteristics of the psoriasis patients are summarized in Table 1. Twenty patients had only skin involvement and 25 were diagnosed with psoriatic arthritis (PsA). Forty-one patients had obesity (BMI ≥30 kg/m^2^) and 4 patients were normal weight but HIV-infected. Twenty-three (51.1%) patients had BMI>35 kg/m^2^, with a mean BMI 41.7 (SD, ±7.2). At the moment of the first dose of vaccine 41 patients (91.1%) were being treated with subcutaneous biologic as monotherapy and 4 patients (8.9%) were on intravenous infliximab in combination with methotrexate. Twenty-one patients (46.7%) were on TNF inhibitors (TNFi) while 24 (53.3%) were on anti-interleukin (ANTI-IL) drugs.

**Table 1:** Baseline characteristics of 45 psoriasis patients.

	***N* (%)**
**Gender**	
Male	28 (62.2)
Female	17 (37.8)
**Age (mean** **±SD)**	59.0 ± 13.1
**BMI (mean** **±SD)**	35.2 ± 8.9
**Educational level**	
Elementary	4 (8.9)
Secondary school	20 (44.4)
High school	13 (28.9)
Degree	8 (17.8)
**Smoking status**	
No	13 (28.9)
Former	15 (33.3)
Yes	17 (37.8)
**Alcohol intake**	
Teetotaler	21 (46.6)
Drinker	5 (11.1)
Former	2 (4.4)
Occasional	17 (37.8)
**Psoriatic arthritis**	
No	20 (44.4)
Yes	25 (55.6)
**Treatment**	
Combination therapy (Infliximab plus Methotrexate)	4 (8.9)
Biologic monotherapy	41 (91.1)
**Biologic type**	
TNFi	21 (46.7)
ANTI-IL 12-23	7 (15.6)
ANTI-IL 17	5 (11.1)
ANTI-IL 23	12 (26.7)
**Remission**	
No	18 (40.0)
Yes	27 (60.0)

BMI, body mass index; TNFi, Tumor necrosis factor inhibitor; IL, interleukin.

Sixty percent of enrolled patients were in complete remission defined by PASI index <3 for only skin psoriasis patients and by minimal disease activity (MDA) criteria for PsA patients (MDA = 5 of the 7 following criteria were met: tender joint count < or = 1; swollen joint count < or = 1; Psoriasis Activity and Severity Index < or = 1 or body surface area < or = 3; patient pain visual analog score (VAS) < or = 15; patient global disease activity VAS < or = 20; health assessment questionnaire < or = 0.5; tender entheseal points < or = 1).

The control cohort consisted of 45 age, sex and BMI matched non psoriasis controls recruited from the staff of Istituti Fisioterapici Ospitalieri (IFO), Rome, Italy.

### Serological response to BNT162b2 at different time points

Table 2 reports the comparative analysis of anti SARS-CoV-2 spike protein GMC of IgG on TP0, TP1, TP2, TP3 and TP4.

**Table 2:** Geometric mean of concentration (GMC) and GM ratios of anti SARS-CoV-2 spike protein IgG at different time points, in psoriasis patients and control group.

		**Controls** ***N* = 45**	**Psoriasis patients** ***N* = 45**	**Mann whitney** ***p*-value**
TP0 (^1st^ dose)	**GMC (95%CI)**	3.88 (3.72–4.06)	5.04 (4.17–6.09)	
TP1 (^2nd^ dose)	**GMC (95%CI)**	50.18 (40.09–62.81)	39.97 (27.22–58.68)	0.319*
	Wilcoxon *p*-value^§^	**<0.001**	**<0.001**	
TP2(4 weeks post ^2nd^dose)	**GMC (95%CI)**	248.22 (224.930–273.91)	273.75 (272.93–274.56)	0.793
	Wilcoxon *p*-value^§^	**<0.001**	**<0.001**	
	**TP2/TP1 ratio GM(95%CI)**	4.95 (4.00–6.11)	6.85 (4.86–9.65)	0.211
TP3 (^3rd^ dose)	**GMC (95%CI)**	88.31 (71.16–109.58)	66.93 (50.22–89.20)	0.066
	Wilcoxon p-value^§^	**<0.001**	**<0.001**	
TP4 (4 weeks post ^3rd^dose)	**GMC (95%CI)**	2050.01 (1,785.68–2353.46)	1,691.89 (1,190.38–2,404.67)	0.324*
	Wilcoxon *p*-value^§^	**<0.001**	**<0.001**	
	**TP4/TP3 ratio GM(95%CI)**	23.22 (18.77–28.71)	25.28 (18.72–34.14)	0.651*
Days between TP0 and ^3rd^dose	**mean** **±SD**	299.91 ± 6.84	196.35 ± 8.65	**<0.001**
	**Median (IQR)**	300 (295.50–304.50)	197 [190–201]	

TP, time point; 95%CI, 95% confidence interval; IQR, interquartile range; ^*^Student' T-test, ^§^Comparisons vs. baseline.

A statistically significant increase in antibody titer to TP1 and TP2, compared to baseline, was observed in both psoriasis patients and control group. No statistically significant difference was found when comparing the antibody response on TP1(*p* = 0.319) and TP2 (*p* = 0.793) of psoriasis patients vs. controls.

The median time elapsed between administration of the first and third dose of vaccine was 300 days for controls (IQR: 295.50–304.50) and 197 (IQR: 190–201) for psoriatic patients.

The GMC declined moderately 5 months after the booster dose (TP3), being 66.93 in psoriatic patients and 88.31 in controls. The comparison between patients and controls did not show statistically significant differences. No cases of seroconversion loss have been observed between the second dose of vaccine and the day of the third dose. The detection performed 1 month after the third dose administration showed a marked increase of the GMC in both patients (1,691.89; 95%CI: 1,190.38–2,404.67) and controls (2,050.01; 95%CI: 1,785.68–2,353.46). The comparison between patients and controls showed GMC levels about 17% lower in patients vs. controls, but this difference was not statistically significant. Moreover, the two groups were not statistically different even in the TP2/TP1 RATIO value (*p* = 0.211).

The four HIV patients showed an antibody response identical to the other psoriasis patients. Only one female patient (Age = 72; BMI = 37,2 kg/m^2^) on combination therapy (infliximab plus methotrexate) did not respond to the double dose of vaccine. Her antibody titer increased from 3.79 AU/mL on TP0 to 5.9 AU/mL on TP2. Nevertheless, the detection performed 4 weeks after the third dose showed a response slightly above the cut-off of 15 AU/mL indicated by DiaSorin^®^ to discriminate responders from non-responders to vaccination. Interestingly, antibody titers on the TP4 detection were significantly lower in patients receiving combination therapy compared to those on biologic monotherapy (Table 3; *p* = 0.037).

**Table 3:** Geometric mean of concentration (GMC) and GM ratios of anti SARS-CoV-2 spike protein IgG at different time points according to different treatment regimens.

		**Combination therapy** **(Infliximab + Methotrxate)** ***N* = 4**	**Biologic monotherapy** ***N* = 41**	**Mann Whitney** ***p*-value**
**TP0 (**^**1st**^ **dose)**	**GMC (95%CI)**	5.80 (2.09–16.04)	4.97 (4.13–5.98)	
**TP1 (**^**2nd**^ **dose)**	**GMC (95%CI)**	17.34 (2.06–145.84)	43.36 (30.11–62.45)	0186*
	Wilcoxon p-value^§^	0.285	<0.001	
**TP2 (4 weeks post** ^**2nd**^**dose)**	**GMC (95%CI)**	106.30 (7.45–1515.73)	300,21 (238.24–378.31)	0.111
	Wilcoxon *p*-value^§^	0.068	<0.001	
	**TP2/TP1 ratio GM (95%CI)**	6.13 (1.29–29.02)	6.92 (4.90–9.79)	0.846*
**TP3 (**^**3rd**^ **dose)**	**GMC (95%CI)**	51.56 (25.18–105.59)	68.65 (54.44–86.56)	0.829*
	Wilcoxon *p*-value^§^	0.068	<0.001	
**TP4 (4 weeks post** ^**3rd**^**dose)**	**GMC (95%CI)**	515.53 (304.66–872.35)	1899.79 (1374.04–2626.70)	0.037*
	Wilcoxon *p*-value^§^	0.068	<0.001	
	**TP4/TP3 ratio GM (95%CI)**	10.00 (7.09–14.10)	27.67 (20.69–37.02)	0.120

TP, time point; 95%CI, 95% confidence interval; ^*^Student' T test, ^§^Comparisons vs. baseline.

Four subjects (all psoriasis patients) showed a positivity to the serological test performed at baseline probably due to asymptomatic COVID-19 infection occurred before the study began, however, a sensitivity analysis excluding the four positive patients did not reveal statistically significant differences in the results obtained (Supplementary Table S2).

### Predictors of antibody response to BNT162b2

The impact of age, gender, BMI and diagnosis of psoriasis on TP4 antibody titers was investigated in all subjects (psoriasis patients and controls) with the GLM (Supplementary Table S3). No variables were significantly associated with the antibody response.

The GLM of antibody titers of psoriasis patients on TP4, identified higher BMI as a significant predictor of a greater antibody response to the third dose of the vaccine (Table 4).

**Table 4:** Generalized linear model (GLM) of IgG at TP4 (4 weeks post ^3rd^dose) in psoriasis patients.

	**Univariate model** **Beta (95%CI)**	***p*-value**	**Multivariate model** **Beta (95%CI)**	***p*-value**
**Age (years)**	−0.003 (−0.030; 0.024)	0.839		
**Gender**				
Female vs. Male	0.250 (−0.464; 0.963)	0.492		
BMI (Kg/cm^2^)	0.039 (0.001; 0.077)	0.044	0.038 (0.002; 0.074)	0.038
**Educational level**				
Elementary	-	-		
Secondary school	−0.806 (−2.056; 0.445)	0.207		
High school	−0.623 (−1.929; 0.683)	0.350		
Degree	−0.389 (−1.788; 1.009)	0.585		
**Smoking status**				
No	-	-		
Former	−0.265 (−1.145; 0.615)	0.555		
Yes	−0.094 (−0.950; 0.762)	0.830		
**Alcohol intake**				
Occasional + drinker vs Teetotaler + former	−0.651 (−1.320; 0.018)	0.057		
**Psoriatic arthritis**				
yes vs. no	−0.537 (−1.219; 0.145)	0.123		
**Treatment**				
Biologic monotherapy vs Combination therapy*	1.304 (0.143; 2.465)	0.028	1.276 (0.166; 2.385)	0.024
**Biologic type**				
TNFi vs. ANTI-IL	−0.444 (−1.129; 0.241)	0.204		
**Remission**				
yes vs. no	−0.196 (−0.904; 0.511)	0.587		

95%CI, 95% confidence interval; BMI, body mass index; TNFi, Tumor necrosis factor inhibitor; IL, interleukin; *Combination Therapy, Infliximab plus Methotrexate.

The same statistical model also demonstrated a stronger antibody response in patients on monotherapy as compared with patients on combination therapy (biologic plus methotrexate). Conversely, no differences were observed when comparing patients on TNFi and those on ANTI-IL treatment.

The impact of additional variables (smoking status, alcohol intake, education level, diagnosis of psoriatic arthritis, remission, type of biologic used) also was assessed on the TP1 response rate by a univariate logistic regression model in the psoriasis patients. None of the included variables was shown to influence the response rate (data not shown).

### BNT162b2 vaccine safety

Although a detailed safety analysis has not been carried out on the study cohort, no local or systemic adverse effects have been reported on the three TPs except for mild pain on the injection site and mild fever mainly after the second dose. No worsening of skin or joint inflammation was observed among the herein reported cases during the weeks following the three doses of vaccine.

### SARS-CoV-2 infection during the study period

No enrolled patients tested positive for nose and throat swabs collected on each TP and none had symptomatic infections during the observational period.

## Discussion

The BNT162b2 vaccine is highly effective and safe, as demonstrated in phase 3 clinical trials, reaching a clinical efficacy of 95% in preventing severe forms of COVID-19 (26). However, real-world data on the efficacy and safety of mRNA vaccines are limited, especially in those patients with chronic inflammatory diseases treated with immunomodulators.

Although patients with psoriasis and psoriatic arthritis do not show an increased vulnerability to SARS-CoV-2, any immune system alteration makes the clinical course of COVID-19 largely unpredictable. In addition, several immunomodulators used in inflammatory disorders have been associated with impaired rates of humoral response to the SARS-CoV-2 vaccine (Table 5).

**Table 5:** Real-world data of COVID-19 vaccine response in psoriasis patients.

**Author (ref.)**	**Study location**	**Sample size**	**Vaccine employed**	**Antibody response**	**Relevant remarks**
Geisen et al. (27)	Germany	6 patients	Moderna mRNA-1273; Pfizer, BNT162b2	IgG titers significantly lower in patients as compared with controls	No specific data related to psoriasis patients. All patients on Biologics. No significant response differences between cDMARDs and biologics
Furer et al. (28)	Israel	165 patients	Pfizer, BNT162b2	Patients response rate 96.9%; IgG titers significantly lower in patients as compared with controls	No specific data related to psoriasis patients. Appropriate immunogenic response in patients on biologic monotherapy and significantly reduced in patients on MTX or on combination therapy (TNFi+MTX) Age >65 associated to a lack of humoral response
Braun-Moscovici et al. (29)	Israel	30 patients	Pfizer, BNT162b2	Patients response rate 86%	No specific data related to psoriasis patients. Treatment with MTX and older age were associated with lower levels of neutralizing IgG.
Al-Janabi et al. (30)	UK	107 patients	Pfizer, BNT162b2 AstraZeneca, ZD1222	Lower response rate in patients as compared with controls	No specific data related to psoriasis patients. Increasing age and using MTX were associated with reduced antibody response. Analysis performed after a single dose of vaccine
Simon et al. (31)	Germany	35 patients	Pfizer, BNT162b2	Lower and delayed response in patients as compared with controls	No specific data related to psoriasis patients. Increasing age was associated with reduced antibody response. No different response between patients on Biologic treatment vs. cDMARDS
Mahil et al. (32)	UK	84 patients	Pfizer, BNT162b2	Patients response rate 78 vs. 100% of controls	Response rates were lower in patients on MTX. Analysis performed after a single dose of vaccine. Results supported by cell-mediated response.
Wieske et al. (33)	Netherlands	~ 150 patients	Pfizer, BNT162b2, Moderna, mRNA-1273, AstraZeneca, ZD1222	Patients response impaired by specific immunomodulators (MTX and TNFi)	No specific data related to psoriasis patients Third vaccination resulted in additional seroconversion
Piros et al. (34)	Hungary	102 patients	Pfizer, BNT162b2, Moderna, mRNA-1273	No significant differences in the median serum level of anti-SARS-CoV-2 antibody were observed between the study population and the control group	Psoriasis patients under biologic treatment. Highest serum level of anti-SARS-CoV-2 antibody was measured in the IL-12/23 inhibitor group. No data about MTX. Evidence of the typical mild adverse effects in the period following the administration of the vaccine
Venerito et al. (35)	Italy	40 patients	Pfizer, BNT162b2	Antibody response not significantly different from matched controls	Trial conducted exclusively on PsA patients in therapy with biologics and DMARDs. MTX use was not associated with a lower anti-SARS-CoV-2 IgG titer. Glucocorticoid use was a predictor of lower immunogenicity
Widdifield et al. (36)	Canada	47.199 patients	Pfizer, BNT162b2, Moderna, mRNA-1273	Overall adjusted vaccine effectiveness of two doses of mRNA-based COVID-19 vaccine against SARS-CoV-2 infection was	Vaccine efficacy is established on PCR test positivity rate.
				84%. No assessment about the effect of immunomodulatorson vaccine efficacy.	
Mahil et al. (37)	UK	67 patients	Pfizer, BNT162b2	100% of seroconversion after the second BNT162b2 dose. No statistically significant difference in spike-specific IgG titres following the second dose between patients receiving immunomodulators and healthy controls. Median titres were numerically lowest in patients receiving methotrexate compared with patients on biologics and healthy controls.	Only 15 healthy controls. Cellular immunity is defined as spike-specific T-cell responses. A lower proportion of participants on methotrexate and biologics had detectable T-cell responses following the second vaccine dose, compared with controls.

Two core topics are driving the scientific debate around the use of COVID-19 vaccines in patients with chronic inflammatory diseases on immunomodulating treatment.

### Risk of psoriasis relapse in response to the vaccine-induced immune stimulation

In this study, we observed no cases of significant worsening of joint and skin manifestations of psoriasis, although at the time of vaccination not all of our patients were in complete remission. Shoenfeld et al. suggested a possible role of vaccines (including COVID-19 vaccines) as triggers for relapse or onset of autoimmune diseases, due to the potential agonism of vaccines on TLRs 7/8 or 9 (65). Although extensive real-world experience is lacking, to date 46 cases of psoriasis relapses following vaccination with COVID-19 have been described in the literature (38–54).

In some rare cases, relapses progressed to particularly severe forms such as erythrodermic or generalized pustular psoriasis. The immunopathogenic mechanisms underlying these exacerbations after vaccination with COVID-19 mRNA vaccines are not yet understood, however according to a recent focus by Watad et al., most of these events should be considered rare and usually responsive to therapy (66).

### Lack of appropriate protective immune response after vaccination

We considered the neutralizing IgG titer anti-SARS-CoV-2 as a proper index of the immune response to BNT162b2.

The present study examined a cohort of patients with at least two factors potentially impairing immune response to vaccines: an immune-mediated disorder characterized by an imbalance of cytokines and lymphocytes such as psoriasis and the treatment with different types of immunomodulators.

Our study population showed an appropriate humoral response to the BNT162b2 vaccine when compared to controls. This is partly in contrast with previous studies reporting impaired response rates and lower antibody titers in patients with various immune-rheumatologic diseases (27–33). However, broad agreement exists around biologics used in psoriasis and psoriatic arthritis minimally affecting vaccine response (34–37).

Importantly, methotrexate negatively affected the antibody response, although firm conclusions around its role as a single agent cannot be drawn, as it was used only in four patients in combination with infliximab and discontinued for 1 week after the vaccination. Nevertheless, methotrexate impairment of the antibody response to vaccines has been widely demonstrated and temporary discontinuation during vaccination is recommended by most scientific societies (67–76).

Approximately 5 months after the second dose we observed a significant decline in antibody titer. Whether that was significant in terms of protection from viral infection cannot be assessed due to the small sample size and the limited follow-up. Nevertheless, none of our patients reported COVID-19 symptoms during the study time. Mahil et al. showed not all patients on immunomodulators were able to develop a T cell response after the second dose vaccination, suggesting a possible faster decay of cell-mediated immunity in this type of patients (37). Conversely, according to Wiskle et al., the reduced antibody response observed in patients receiving immunomodulators may not result in a clinically significant short-term loss of protection, being these medications unable to affect memory B-cell function. Nevertheless, the same authors reported an increased rate of seroconversion after the third dose of vaccine in patients treated with specific immunomodulators (33).

Four weeks after the third dose, psoriasis patients showed antibody levels ~10-fold higher than those achieved after the second dose, with no significant differences compared to control subjects. In accordance with the results of Wiskle et al., our GLM model showed methotrexate used in combination with TNFi to have a slight negative impact on the antibody response even after the third dose of vaccine (33).

Obesity is an extensively studied metabolic disorder characterized by chronic low-grade inflammation potentially impairing both humoral and cell-mediated immune function.

The combination of these phenomena could explain both the poor prognosis of obese patients in COVID 19 infection, but also an abnormal response to the vaccine (76, 77). There is ample evidence to support a lack of efficacy in the antibody response of obese people to common vaccines such as influenza, rabies and hepatitis B (78, 79).

Diversely, the multivariate analysis we performed revealed increased BMI to favorably influence the antibody response. In line with this interesting finding, we have previously demonstrated that obesity was unable to influence the antibody response to BNT162b2 in psoriatic patients after two doses of BNT162b2 vaccine as also highlighted by Cristaudo et al. (59) and Pellini et al. (80).

Interestingly, neither the type of biological treatment (TNFi vs. ANTI-IL) nor the clinical condition at the time of vaccination (remission vs. active disease) influenced the humoral response to vaccine. Patients with an additional immunological risk factor, such as HIV infection, also had satisfactory antibody responses to the vaccine. Our study has limitations, mainly owing to the small number of patients enrolled and the duration of follow-up, therefore, the multivariate analyses we performed could not have provided significant results. Nevertheless, we confirmed the safety of BNT162b2, both in terms of typical vaccine adverse effects and risk of psoriasis relapse. Three doses of mRNA vaccine elicited a vigorous anti-Spike response, presumably providing effective protection from SARS-CoV-2 infection, as highlighted by the lack of symptomatic COVID-19 in our study population. In conclusion, the risks-benefits balance of BNT162b2 vaccine in a psoriatic population treated with immunomodulators is strongly in favor of the benefits. Thus, we strongly support the implementation of the third vaccination for selected patients with immune-mediated inflammatory disorders receiving immunomodulators as a useful measure to improve immune response to the vaccine and supposedly the level of protection against SARS-CoV-2. Further targeted studies will be needed in order to assess in patients treated with immunomodulators the persistence of antibody protection and the need for subsequent booster vaccinations.

## Data availability statement

The original contributions presented in the study are included in the article/Supplementary materials, further inquiries can be directed to the corresponding author.

## Ethics statement

The studies involving human participants were reviewed and the study was approved by the IRCCS Central Ethical Committee of Regione Lazio in January 2021 (Prot. N-1463/21). The patients/participants provided their written informed consent to participate in this study.

## Author contributions

All authors listed have made a substantial, direct, and intellectual contribution to the work and approved it for publication.

## Funding

This research was funded by the Italian Ministry of Health (RC 2022).

## Conflict of interest

The authors declare that the research was conducted in the absence of any commercial or financial relationships that could be construed as a potential conflict of interest.

## Publisher's note

All claims expressed in this article are solely those of the authors and do not necessarily represent those of their affiliated organizations, or those of the publisher, the editors and the reviewers. Any product that may be evaluated in this article, or claim that may be made by its manufacturer, is not guaranteed or endorsed by the publisher.

## Footnotes

^1^ https://www.diasorin.com/en/immunodiagnostic-solutions/clinical-areas/infectious-diseases/covid-19
