# Supplementary material for: Immunogenicity of three doses of anti-SARS-CoV-2 BNT162b2 vaccine in psoriasis patients treated with biologics
Source: Front Med (Lausanne). 2022 Sep 6;9:961904. doi: 10.3389/fmed.2022.961904 (PMC9485492; doi:10.3389/fmed.2022.961904)
Supplement: Supplementary file 1 [file Table_1.DOCX]

| **Table S1.** Case-matched analysis comparing psoriasis patients vs control group. | | | |
| --- | --- | --- | --- |
|  | **Controls N=45**  **N (%)** | **Psoriasis patients N=45**  **N (%)** | **p-value** |
| **Age (mean±SD)** | 52.86 ±7.14 | 59.01 ±13.12 | ***0.014**** |
| **Gender** |  |  |  |
| **M** | 20 (44.4) | 28 (62.2) | *0.091*** |
| **F** | 25 (55.6) | 17 (37.8) |  |
| **BMI (mean±SD)** | 24.83±3.36 | 35.21±8.93 | ***<0.001°*** |
| **<35** | 44 (97.8) | 22 (48.9) | ***<0.001***** |
| **≥ 35** | 1 (2.2) | 23 (51.1) |  |
| BMI: body mass index, *Mann-Whitney test. **Chi2 test. °T-Student test. | | | |
