# Supplementary material for: Immunogenicity of three doses of anti-SARS-CoV-2 BNT162b2 vaccine in psoriasis patients treated with biologics
Source: Front Med (Lausanne). 2022 Sep 6;9:961904. doi: 10.3389/fmed.2022.961904 (PMC9485492; doi:10.3389/fmed.2022.961904)
Supplement: Supplementary file 2 [file Table_2.DOCX]

| **Table S2.** Sensitivity analysis excluding the 4 subjects with positive antibody titer at baseline. | | | | |
| --- | --- | --- | --- | --- |
|  |  | **Controls**  **N=45** | **Psoriasis patients**  **N=41** | **Mann-Whitney**  **p-value** |
| **TP0(^1st^ dose)** | **GMC (95%CI)** | 3.88 (3.72-4.06) | 4.24 (3.87-4.65) |  |
| **TP1(^2nd^ dose)** | **GMC (95%CI)** | 50.18 (40.09-62.81) | 37.66 (26.36-53.80) | *0.186** |
|  | *Wilcoxon p-value^§^* | ***<0.001*** | ***<0.001*** |  |
| **TP2(4 weeks post ^2nd^dose)** | **GMC (95%CI)** | 248.22 (224.930-273.91) | 257.88 (190.83-348.49) | *0.548* |
|  | *Wilcoxon p-value^§^* | ***<0.001*** | ***<0.001*** |  |
|  | **TP2/TP1ratio GM(95%CI)** | 4.95 (4.00-6.11) | 6.85 (4.82-9.72) | *0.192* |
| **TP3(^3rd^dose)** | **GMC (95%CI)** | 88.31 (71.16-109.58) | 65.91(48.64-89.32) | *0.067* |
|  | *Wilcoxon p-value^§^* | ***<0.001*** | ***<0.001*** |  |
| **TP4(4 weeks post  ^3rd^dose)** | **GMC (95%CI)** | 2050.01(1785.68-2353.46) | 1731.8 (1181.25-2538.87) | *0.420** |
|  | *Wilcoxon p-value^§^* | ***<0.001*** | ***<0.001*** |  |
|  | **TP4/TP3 ratio GM(95%CI)** | 23.22 (18.77-28.71) | 26.27(18.97-36.38) | *0.535** |

TP: time point, 95%CI: 95% confidence interval, *Student’ T test, ^§^Comparisons vs baseline
