# Supplementary material for: Immunogenicity of three doses of anti-SARS-CoV-2 BNT162b2 vaccine in psoriasis patients treated with biologics
Source: Front Med (Lausanne). 2022 Sep 6;9:961904. doi: 10.3389/fmed.2022.961904 (PMC9485492; doi:10.3389/fmed.2022.961904)
Supplement: Supplementary file 3 [file Table_3.DOCX]

**Table S3.** Generalized linear model (GLM) of IgG at TP4 (4 weeks post ^3rd^dose) in all subjects.

|  | **Univariate model** | |
| --- | --- | --- |
|  | **Beta (95%CI)** | **p-value** |
| **Age (in years)** | -0.003  (-0.020; 0.015) | *0.762* |
| **BMI (Kg/cm^2^)** | 0.016  (-0.006; 0.038) | *0.156* |
| **Gender** |  |  |
| ***female vs male*** | 0.169  (-0.205; 0.544) | *0.376* |
| **Psoriasis patients** |  |  |
| ***cases vs controls*** | -0.192  (-0.566; 0.182) | *0.314* |

BMI: body mass index, 95%CI: 95% confidence interval
